# Recent advances of therapeutic targets based on the molecular signature in breast cancer: genetic mutations and implications for current treatment paradigms

**DOI:** 10.1186/s13045-019-0725-6

**Published:** 2019-04-11

**Authors:** Zeinab Safarpour Lima, Mostafa Ghadamzadeh, Farzad Tahmasebi Arashloo, Ghazaleh Amjad, Mohammad Reza Ebadi, Ladan Younesi

**Affiliations:** 1 Shahid Akbar Abadi Clinical Research Development Unit (ShCRDU), Iran University of Medical Sciences (IUMS), Tehran, Iran; 2 Departement of Radiology, Hasheminejad Kidney Centre (HKC), Iran University of Medical Sciences, Tehran, Iran; 3 Clinical Research Unit, Afzalipour Hospital, Kerman University of Medical Sciences, Kerman, Iran; 4 Shohadaye Haft-e-tir Hospital, Iran University of Medical Sciences (IUMS), Tehran, Iran

**Keywords:** Breast, New drug, Chromosome, Molecular patterns, Mutations

## Abstract

Breast cancer is the most common malignancy in women all over the world. Genetic background of women contributes to her risk of having breast cancer. Certain inherited DNA mutations can dramatically increase the risk of developing certain cancers and are responsible for many of the cancers that run in some families. Regarding the widespread multigene panels, whole exome sequencing is capable of providing the evaluation of genetic function mutations for development novel strategy in clinical trials. Targeting the mutant proteins involved in breast cancer can be an effective therapeutic approach for developing novel drugs. This systematic review discusses gene mutations linked to breast cancer, focusing on signaling pathways that are being targeted with investigational therapeutic strategies, where clinical trials could be potentially initiated in the future are being highlighted.

## Introduction

Several risk factors was recognized to be associated with breast cancer development including age, hormonal, reproductive, menstrual history, alcohol, radiation, hereditary factors, obesity, etc. [1]. Among these risk factors, age is the biggest risk factor for developing breast cancer followed by a positive family history. Based on data presented in literatures, previous studies discovered several features of inherited mutations in the genes. It was estimated that about 10–30% of breast cancer cases are related to hereditary factors, also 5–10% of breast cancers were detected with strong hereditary factors, while between 4 and 5% of these cases was identified by mutations in high-penetrant genes [2].

BRCA1 and BRCA2 have been known as regulators of DNA repair, transcription, and cell cycle in reply to DNA damage. BRCA1 and BRCA2 genes are the most commonly mutated genes that are associated with high breast cancer risk [3, 4]. It has been reported that 60% of hereditary breast cancers can be related to germline mutations in either of these genes [5]. The number of genes are associated with multiple cancer syndromes for example phosphatase and tensin homolog protein (PTEN) (Cowden syndrome), TP53 (Li-Fraumeni), STK11/LKB1 (Peutz-Jeghers), ataxia telangiectasia (ATM) (Louis-Bar Syndrome), and NBS1 (Nijmegen breakage syndrome), but other genes associated with hereditary breast cancer are emerging. Cancer predisposing genes can be classified as high-penetrant genes including BRCA2, BRCA2, TP53, STK11, and CDH1 [6–9]. On the other hand, the majority of gens can be categorized as moderate-penetrant genes in most of the breast cancer cases including CHEK2, ATM, CDH1, NBS1, BRIP1, PALB2, BARD1, RAD50, and RAD51, which are frequently mutated in the general population and contribute in developing of breast cancer [3, 10, 11].

The present study try to focus on the spectrum of mutations, polymorphisms, and variants in each gene which are linked to breast cancer as well as how it contributes to the disease.

## Current aspects and personalized medicine

There are several cancer syndromes in which the alleles are located in a predominant autosomal dominant fashion and thus lead to a high risk of neoplasm. In addition, non-genetic factors have been revealed to be implicated in mutation or other genetic changes. Together with genetic changes in tumors, a variety of inherited genetic changes in the genes are involved in drug metabolism, which are capable of affecting therapeutic responses (e.g., increasing toxicity of the drug). Advances in “pharmacogenomic” science lead to providing a specific treatment based on individual genetic information [12]. In this regard, personalized medicine open an opportunity for applying the correct therapeutic strategy at the appropriate, with the least toxic or non-toxicity, and this treatment should be appropriate for a suitable patient at the right time, where treatment will rely on genetic changes in everyone’s cancer. As a matter of fact, future-oriented attitudes in the medical arena should be taken into consideration because we need to keep in mind that genetic testing will be able to help decide on better therapies where the patient does not respond to a certain treatment, because this approach can avoid receiving medications that are not likely to be helpful.

Given the personalized medicine, information about genetic changes in the tumor can help to elucidate the right therapeutic decision. The completion of the human genome project and the continuous advancement in genetic research will facilitate the diagnosis and treatment of many diseases. Genomic studies developed to increase the medical awareness and ability to produce, analyze, and interpret effective genetic data. However, their effects on clinical performance occur at a much slower rate [13, 14]. Scientists now realize that changes that occur in a person’s cancer may not occur in other people with the same type of cancer, while the same cancerous changes may be found in a variety of cancers.

We now learn more about genomic changes (change in number of copies, deletion, mutations, single-nucleotide polymorphism) and the correlation of these mutations with many types of cancers. These communication studies help determine who is at risk for cancer [15]. It is hoped that the genomic cancer will expand with the use of whole-genome sequencing (WGS), DNA sequencing technology, and cancer cell analysis enables researchers to clearly reveal new genetic changes associated with cancers, which is likely to be very beneficial for the development of personalized medicine. Providing an integrated analysis and systematic characterization of key genomic mutations in different kinds of breast cancer (BC) subtypes can lead to advances in the use of favorable diagnostic, therapeutic, and preventative strategies. Cancer can be considered to be a genomic disease that each tumor has a set of specific genetic alteration. In-depth understanding of the genetic alteration and molecular mechanisms that underlie regulation of gene expression profiles in BC cells leads to favorable effective therapeutic approach that is dedicated to the genetic profile of each person.

## High-penetrant gene

### BRCA1 and BRCA2

BRCA1 gene has large coding sequences on chromosome 17q, consisting of 22 exons coding exons [16, 17]. BRCA2 gene consist of 26 coding exons on chromosome 13q [18]; a large number of mutations of both mentioned genes were characterized worldwide. To the best of knowledge, many mutations have been described in the BRCA1 gene (1800 mutations) and the BRCA2 gene (2000 mutations) based on the data presented in BC Information Core that is available at https://research.nhgri.nih.gov/bic/resources.shtml [19]. As indicated, different mutations are capable of conferring risks of ovarian cancer or BC including missense mutations, intronic changes, deletions, large rearrangements, and small in-frame insertions [19, 20]**.**

BRCA1 and BRCA2 are postulated to play a crucial role in maintaining DNA integrity and are tumor suppressor genes. Inherited mutation in the BRCA1 and BRCA2 genes is caused by hereditary BC.

Current evidence suggests that specific inherited mutations of BRCA1 and BRCA2 are attributed to an enhanced risk of female BC and ovarian cancer (OV). These two genes are regarded to be approximately responsible for 6–7% of BCs, and 10% OV these rates vary between different populations [3] and have been linked to higher risks of different types of cancer [4]. However, not all breast and breast-ovarian cancer families carry a mutation in BRCA1 or BRCA2. Breast and ovarian cancers related to BRCA1 and BRCA2 mutations tend to occur more often in younger ages than their nonhereditary counterparts.

To the best of our knowledge, the majority of mutations of the BRCA1/BRCA2genes in breast and/or ovarian cancer families are point mutations or small insertions and deletions spread over the coding sequence and splice site junctions. It is noteworthy that large rearrangements in BRCA1and BRCA2 accounted for less than 1% of evaluated patients suffering from hereditary breast and ovarian cancer [21]. All of these mutations are capable of shortening BRCA1 protein, leading to failing to its physiologic function [9]. Furthermore, aggressive triple-negative breast cancer (TNBC) has also been associated with sporadic mutations in BRCA1 [22, 23]. In addition, 853 unique mutations, polymorphisms, and variants in the BRCA2 genes were also reported [9].

Male BC is an uncommonly occurred malignancy, accounting for 1% of all male cancers and 0.65% of all breast tumors [24, 25], which remarkably diagnosed at an older age in comparison with female (mean age 67 years). It is worth noting that 10% of males with BC has found to attributed to a genetic predisposition with BRCA2 as the most frequent genetic mutation [26, 27], accounting for 4–40% of hereditary BC in men [28]. Accumulating evidence indicates that BRCA1/2 m mutations can be considered as clinically appropriate and relevant for determining subtypes of HER2-positive*/*HER2-negative BC [20, 29, 30]. Ample evidence has indicated that BRCA1/2 m testing can hold great promise for identifying subjects with breast cancer who receive effective platinum-based chemotherapy and PARP inhibitors-based therapy [20]; because subjects as a homogeneous group with breast cancer exhibited to be negative for predictive biomarkers including estrogen-receptor-positive (or ER+), PGR progesterone receptor, and HER2 gene amplification or HER2 protein overexpression, when underwent chemotherapy because of either their lack of molecular marker for the targeted therapy or lack of specific therapeutic approach for triple-negative breast cancer (TNBC) subtypes [31]. It is worth noting that loss-of-function mutations in BRCA1/2 has been revealed for approximately 20% of unselected TNBC patients, holding a great promise for the development of accurate therapeutic approach [32]. We mentioned findings obtained with PARPis, in preclinical phases of development for which there is a growing body of evidence of strengthening effects of platinum in vivo [33–35], where synergism between PARPis and platinum compounds has been revealed in BRCA mutated breast cancer [36, 37]. Efficacy of the veliparib alone and its combination therapy with carboplatin has been currently suggested in subjects with gBRCA1/2 mutations-associated metastatic breast cancer [38]. A phase II trial (BROCADE, NCT01506609) evaluated veliparib (ABT-888) in combination with paclitaxel and carboplatin vs placebo, where findings revealed a 77.8% vs. 61.3% response rate [36]. In addition, benefit of veliparib combinations has been potentially indicated by a phase III randomized prospective trial (NCT 02163694) for patients suffering from BRCA-associated metastatic BC. Olaparib monotherapy offer opportunities for the development of therapeutic targets in patients suffering from HER2-negative metastatic breast cancer and germline BRCA1/2 mutations, where exhibited to be beneficial over standard therapy [39]. In a phase 3 trial, talazoparib was used in comparison with other therapeutic strategies of physician including capecitabine, gemcitabine, eribulin, and vinorelbine for treatment of advanced breast cancer and a germline BRCA mutation, where talazoparib use was found to be linked to prolonged PFS, and a remarkable delay in time was revealed for deterioration in global health status/quality of life [40].

PARPi and other therapy that are being assessed in clinical trial studies in breast cancer associated with BRCA1/2m, including rucaparib, veliparib, niraparib, talazoparib, and olaparib, are summarized in Table 1 based on the date adopted from clinicaltrials.gov.

**Table 1:** Therapeutic approaches for breast malignancies in clinical trials in subjects with BRCA1 and BRCA2 mutations. Tables and data are adopted from clinicaltrials.gov. (https://clinicaltrials.gov/)

Conditions (Title)	Interventions of clinical trial	Study type	Study completion
Metastatic breast cancer	• Drug: Placebo, veliparib, and carboplatin	Interventional, phase 2	2019
• BRCA1 and BRCA2 mutation carrier with BC • Hereditary BC/OC with BRCA1 and BRCA2	• Drug: Letrozole, and placebo	Interventional, phase 3	2022
• Recombinant hCG • BRCA1 and BRCA2 mutation in BC	• Drug: Ovitrelle, and recombinant Hcg	Interventional phase 4	2021
• OC, BC, and prostate cancer	• Drug: Olaparib	Interventional, phase 2	2018
• BRCA1 and BRCA2 mutation carrier • BC and metastatic BC, and OC	• Drug: Rucaparib (CO-338; AG-014699 or PF-01367338) • Expression evaluation	Interventional, phase 2	2015
• BC metastatic • BRCA1 and BRCA2 mutation carrier	• Drug: Olaparib therapy, and chemotherapy with doctor’s choice	Interventional, phase 3	2019
• BRCA1 and BRCA2 mutation carrier in BC	• Biological: Therapeutic estradiol • Drug: Deslorelin, therapeutic testosterone • Deslorelin combined with low-dose add-back estradiol and testosterone	Interventional, phase 2	2019
• BRCA1 and BRCA2 mutation carrier in BC	• Supplement: S0812: cholecalciferol supplementation	Interventional, not applicable	2017
• BC • Metastatic BC • BRCA1 and BRCA2 mutation	• Drug: ABT-888 and temozolomide	Interventional, phase 2	2020
• Human EGF2 negative carcinoma of breast • BRCA1 and BRCA2 mutation	• Drug: Niraparib as neoadjuvant therapy	Interventional, phase 1	2020
• Breast neoplasms	• Drug: Dexamethasone, trabectedin	Interventional, phase 2	2011
• Breast malignancy • BRCA1 and BRCA2 mutation carrier	• Drug: Talazoparib (BMN 673)	Interventional, phase 2	2018
• Breast neoplasms • BRCA1 and BRCA2 mutation carrier	• Drug: Talazoparib (BMN 673): a PARP inhibitor • Drug: Physician’s choice	Interventional, phase 3	2019
• Advanced BC • HER2/Neu negative • TNBC	• Drug: Talazoparib Tosylate	Interventional, phase 2	2019
• BC: Stage IV • OC • BRCA1 and BRCA2 mutation	• Drug: Carboplatin, eribulin, and veliparib	Interventional, phase 2	2020
• Basal-like breast carcinoma • BRCA1 and BRCA2 mutation	• Drug: Veliparib • Laboratory evaluation and biomarker assessment	Interventional, phase 1	2017
• Atypical ductal breast hyperplasia • BRCA1 and BRCA2 mutation carrier	• Metformin hydrochloride as drug • Placebo	Interventional, phase 3	
• Atypical ductal breast hyperplasia • BRCA1 and BRCA2 mutation	• Dietary supplement: curcumin • Biomarker evaluation	Interventional, not applicable	2019

Accumulating evidence suggests that BRCA1/2 mutations can be considered as predictor of the response to PARPi in patients suffering from metastatic breast cancer. It is worth noting that PARPi has been currently confirmed for breast cancer therapy, which were clinically evaluated; on the other hand, olaparib has been also introduced for BRCA1/2 mutations carriers with HER2-negative metastatic breast cancer.

An increasing body of evidence indicated that platinum-based regimen can be used for BRCA1/2 mutation carriers with TNBC, for whom cancer chemotherapy with anthracycline cannot be performed. However, it should be taken into account that standard chemotherapy is likely to be applied as long as more information on the effect of platinum chemotherapy be available for subjects with positive hormone receptor breast cancer carrying BRCA1/2mutations [20].

### STK11/LKB1

The serine/threonine kinase gene (STK11/LKB1) is a substantial tumor suppressor gene, which acts as an energy metabolic sensor and is involved in cell polarity regulation and mediation of apoptosis. The STK11 gene plays a key role in cell proliferation via many targets, where a requirement has been suggested for the tumor suppressor function of this kinase and/or STK11 catalytic activity [41–43]. One of the most important downstream targets of LKB1 is the energy sensor AMP-activated protein kinase (AMPK). LKB1 is capable of phosphorylating and activating the AMPK [44, 45]**.** Phosphorylation of AMPK led to activation of TSC1/TSC2, suppression of the mTOR activity, and dephosphorylation of mTOR effectors, S6K and 4E-BP1, which are capable of regulating initiation of protein translation [46]. As a matter of fact, LKB1 is considered as a regulator of many pathways implicated in cell growth and metabolism. In addition, mTOR plays an important role in integrating various cell signals for regulating cell growth [47]. The mTORC1 signaling pathway has been identified to be initially downregulated by kinase activity of STK11 protein, and phosphorylation of the major downstream targets (e.g., S6K1 and S6) can be also prevented by STK11 protein. Current evidence suggests that phosphorylation of S6K1 and S6 can contribute to the inhibition of tumorigenesis and cell proliferation [41, 48, 49]. MTOR overactivation has been also explained to be related to hamartomatous tumor growth, when STK11 inactivation is occurred in mic [50].

Germline mutations in this gene cause Peutz-Jeghers syndrome (PJS) that is subsequently associated with breast cancer [9]. Current evidence suggests that patients with PJS show a 54% risk of developing BC in comparison with the healthy individuals [51]. STK11/LBK1 mutations have been detected to be related to estrogen-receptor positivity, which may work as causative factor of breast cancer in susceptible people. Somatic mutations of LKB1was detected in sporadic pulmonary, breast, pancreatic, and biliary cancers and melanomas. Recent studies have indicated that mutations on threonine kinase gene (on 19p) play a crucial role in developing the PJS. This syndrome has been shown to be responsible for the high susceptibility to many cancers, especially over age 60. Mutations of a single allele in LBK1 may be responsible for an aggressive BC which can lead to decreasing survival time [52].

Based on the data presented by Juan et al. (2014), downregulation of LKB1 has been found to be linked to markers involved in BC prognosis such as decrease of ER/PR, E-cadherin, and HMW-CK. Knockdown of endogenous LKB1 has been shown to contribute to dysregulation of cell polarity and invasive phenotype of BC cells [53]**.**

It has been recently indicated that two novel missense mutations of STK11 are involved in disruption of STK11 function and an impairment of serine/threonine-protein kinase STK11 activity. Therapeutic strategies aimed at targeting the downstream targets of the mTOR signaling pathway common to S6K1 and S6 are considered as potential therapeutic targets against STK11-related cancer.

In addition, in order to reveal new tumor vulnerabilities following LKB1 loss, profiling of the intracellular and extracellular tumor environments would need to determine LKB1 features [54]. Preclinical investigations (e.g., xenografts, cell lines, and GEMMs) have suggested that highly effective therapies will be achieved by targeting LKB1 vulnerabilities and metabolic therapies [54]. Lkb1/Pten-deficient genetically engineered mouse model (GEMM) has been shown to be correlated with enhanced level of PD-L1 expression and lung squamous cell carcinoma [55].

Immunotherapies have a considerable therapeutic impact on cancer patients with melanoma and NSCLC. Anti-PD-1 and PD-L1 therapies have been suggested as potential therapeutic against tumor recurrence and/or metastasis in LKB1-mutant SCC to target TPCs after debulking tumor with a targeted inhibitor or surgery [54–56].

It is worth noting that targeting LKB1 tumors with specific chemotherapy or metabolic therapies are required in preclinical trials, which cancer cell line encyclopedia (CCLE) may be clinically helpful to offer clues [57]

Based on the previous findings [58–60], KRAS inhibitor or epigenetic regulators combined with targeted therapies or energy stress agents may hold great promise for LKB1/KRAS^G12C^ or BRG1/LKB1 co-mutated cancer. More efficacious therapeutic options can be achieved for developing personalized medicine in patients with LKB1 mutations by defining vulnerabilities and their appropriate targeting drugs as well as preforming novel therapeutic strategies in both animal and cell-culture-based models such as organoid models and 2D and 3D culture systems [54]. In addition, orthotopic patient-derived xenograft (PDX) models and GEMMs can be used for providing LKB1 vulnerabilities—targeting therapies in imaging-based preclinical [61–64].

### TP53

TP53 gene at17p13 is responsible for producing TP53 tumor suppressor protein that is responsible to various stress signals and suppression of cellular transformation via mediating cell-cycle arrest, the cellular response against oncogenic stress, and cell repair and apoptosis. TP53 is related to Li-Fraumeni syndrome that is linked to onset of a many malignancies such as BC, brain tumors, leukemia, and lymphoma, and sarcomas, as well as melanoma, lung cancer [8, 65, 66]**.** These small mutations are located in exons 5–8, spanning the DNA-binding domain of the protein [67]. TP53 mutations are considered to be associated with development of 20–40% of BCs, and 90% of cases suffering from metastatic BC in the brain showed TP53 mutation [68]. TP53 mutation has an independent prognostic value in breast cancer, while its predictive value is debated [69, 70]. It has been indicated that a single-nucleotide germline mutational alteration at exon 10 codon 337 of TP53 (CGC to CAC) is responsible for a change in arginine to histidine (R337H) that is associated to early onset BC [71–74]. Based on the current evidence, overexpression of missense mutant p53 was identified in different kinds of cancers and the tumor-promoting GOF activities have been also demonstrated, indicating its potential for targeted therapy in cancers [75].

Regarding the pharmacological strategies, many mutant p53-targeting agents have been provided by different approach over the past decade, where mutant p53-targeting agents in combination with chemotherapeutic drugs (e.g., p53–Mdm2 inhibitors) hold promise for synergies. Based on the data presented in the literatures, rational design using structural knowledge and random screening chemical libraries has been resulted in developing small molecule reactivators of mut p53 proteins. Achieving an attractive pharmacological approaches need to address chemical characteristics of hit compounds and antitumor efficacy; however, these approaches for mutant p53 are challenging because of heterogeneity of mutant p53 and requiring restoration of correct folding in a mutant protein [75].

Y220C-targeting compounds such as PK083 and PK7088 have been designed [76]. PK7088 indicated potential as targets for triggering apoptosis and Y220C-dependent cell cycle arrest of tumorous cells [77].

PK11007 has been revealed to contribute to the inhibition of both the growth and migration and induction of apoptosis in p53-mutated breast cancer cell lines, indicating a potential strategy for subgroup with triple-negative breast cancer [78]. The investigations on reactivation of mutant forms of p53 by using NSC319726 (p53 ^R175H^), PK7088, PK083, PK5174 (p53 ^Y220C^), PRIMA-1, and PRIMA*-*1^Met^ are likely to yield the most effective targeting of mutant p53 in [75, 79].

PRIMA-1 and PRIMA-1^Met^ has been described to be able to restore conformation of wild-type mutant p53, where they have been found to be more effective at inhibiting tumor growth and inducing apoptosis in tumor cells in mice mode. PRIMA-1 has been found to be implicated in reactivating mutant p53 by covalent binding of MQ per se, therefore, can serve as an approach for designing mutant p53-targeting anticancer drugs [75]. In addition, it has been revealed that PRIMA-1 is capable of inhibiting growth of mut p53 breast cancer cells [80, 81]. APR-246 has been further investigated previously by preclinical models, which exhibited an inhibitory effect on the cell proliferation in breast cancer. Importantly, APR-246 in combination with eribulin has been suggested to be considerably effective at inhibition of synergistic cell growth in six different p53-mutated BC cell lines [82]. Response to APR-246 has been also revealed to be linked to mutant p53 BC cells or high protein levels as a predictive biomarker for this response [83].

APR-246 has been clinically evaluated (phase I) in hematologic and prostatic neoplasms [82]; however, it is undergoing clinical trials in subjects suffering from some malignancies (Table 2), while is not currently evaluated in patients with breast cancer.

**Table 2:** APR-246 used in clinical trial among subjects suffering from some malignancies; Tables and data are adopted from clinicaltrials.gov. (https://clinicaltrials.gov/)

Status of trial	Conditions (Title)	Interventions of clinical trial	Study type	Study completion time
Not yet recruiting New	• Treatment of TP53 mutant myelodysplastic Syndromes (MDS)	• Drug: APR-246 + azacitidine and Azacitidine	Interventional, phase 3	2020
Recruiting	• Esophageal carcinoma	• Drug: APR-246	Interventional, phases 1 and 2	2023
Recruiting	• Myelodysplastic syndrome with gene mutation • Acute myeloid leukemia with gene mutations • Myeloproliferative neoplasm • Chronic myelomonocytic Leukemia	• Drug: APR-246 and Azacitidine • Combination therapy with APR-246 and Azacitidine	Interventional, phases 1 and 2	2021
Recruiting	• myeloid neoplasms with TP53 mutant • Acute myeloid leukemia • Chronic myelomonocytic leukemia	• Drug: APR-246 and Azacitidine	Interventional, phases 1 and 2	2021
Active	• BRAF V600 mutant melanoma	• Drug: APR-246 and Dabrafenib	Interventional, phases 1 and 2	2018
Active	• P53 activation in platinum resistant serous advanced OC	• Drug: APR-246 and pegylated liposomal doxorubicin hydrochloride (PLD)	Interventional, phase 2	2019
Completed	• Prostate cancer or refractory hematologic cancer	• Drug: APR-246	Interventional, phase 1	2010
Active	• Platinum sensitive recurrent advanced serous OC (mutated p53)	• Drug: APR-246 and Carboplatin and PLD	Interventional, phases 1 and 2	–

COTI-2, a third-generation thiosemicarbazone derivative, has been provided by CHEMSAS®, a multi-staged computational platform. COTI-2 has been revealed to have an effective anti-proliferative activity function in many human cancer cell lines including tumors for which cetuximab and erlotinib have been applied for providing treatments. In p53 mutant triple-negative breast cancers, COTI-2 has been described to be potentially applicable in providing therapeutic strategy for patients with this subform [83, 84].

Anticancer activity of COTI-2 has been found in many kinds of human tumor cell lines and MDA-MB-231 Xenograft model for breast cancer. COTI-2 has been found to be involved in caspase signaling cascade, resulting in apoptosis in tumorous cells. It has been suggested that the use of COTI-2 is safe and well-tolerated in vivo [84]. COTI-2 phase-I clinical is currently undergoing for gynecologic malignancies and head and neck squamous cell carcinoma that is available on https://clinicaltrials.gov/ct2/show/NCT02433626?cond=COTI%E2%80%912&rank=1.

### CDH1

The E-cadherin gene (CDH1, OMIM192090) on chromosome 16q is responsible for encoding the E-cadherin [85], which has been demonstrated to play a key role in cellular adhesion, cell motility, differentiation, growth, migration, and signaling [86] as well as to function as a tumor suppressor of BC [87]. Carriers of CDH1 gene mutation are at lifetime risk of BC on the order of 40% to 50% [88].

CDH1inactivation not only is directly associated with germline or SM but is also linked to loss of heterozygosity (LOH) and promotor hypermethylation. CDH location of the chromosome 16 is mostly related to LOH and loss of tumor suppressor function in human breast cancer [88]. E-cadherin downregulation is most commonly associated with LOH or promoter hypermethylation in breast cancers.

A growing body of evidence indicated that LOH at the long arm of chromosome 16 is involved in carcinomas in breast. LOH is known as a major mutation for the E-cadherin alleles in developing of lobular breast cancers [89].

It has been shown that individuals with germline mutation are at risk of lobular breast cancer and colorectal cancer [90]. Moreover, women carriers are at lifetime risk of 40–54% of lobular breast cancer [91, 92].

Among subjects with invasive lobular breast cancers, e-cadherin was not expressed [93, 94], whereas smaller rate allocated to the atypical expression. This subtype is mostly related to LOH of the wild-type allele of CDH1. The ductal subtype is recognized to show high heterogeneity, with lower frequency [87, 95].

Invasive lobular breast carcinoma was shown to be typically E-cadherin-negative and inactivating mutations has been frequently found along with LOH of the wild-type allele of CDH1. Moreover, somatic CDH1 mutations were reported in breast cancer, lobular carcinoma in situ (LCIS). It is worth noting that most of somatic mutations are due to premature stop codons as a consequence of insertions; subjects with LCIS have shown LOH for 16q22.1, and under-expression of E-cadherin, as well as SMs in the CDH1 in LCIS [96]. Novel CDH1 germline mutations have been recently revealed in patients suffering from LBC without family history for gastric cancer. On the other hand, CDH1 germline mutations have been revealed to be capable of affecting hereditary lobular breast cancer as an independent-HDGC syndrome [97]. Nevertheless, longtime follow-up is needed for preventing a secondary diffuse gastric cancer.

Increasing evidence suggests that a family history of multiple ILC at younger ages and/or history of early-onset bilateral ILC can be markedly attributed to CDH1 germline mutations [98].

The rates of women with lobular type of BC are unclear, as histologic findings have not been specified. In addition, based on the available data, no higher age limit was reported for patients suffering from family ILC; on the other hand, BRCA1/2 mutations were not also excluded from all patients.

Prescribing CDH1 germline analysis should be taken in to consideration for subjects with pathological confirmation of early-onsets ILC, for both personal and familiar background [98].

Subjects with CDH1mutations are advised to check annual mammograms and breast MRIs starting at age 35 years due to high lifetime risk of developing LBC [99, 100]. CDH1germline mutations have been suggested to be also exclusive with BRCA1/2 alterations. In addition, the risk of ILC has been defined to be very close to the risk of BC in subjects harboring BRCA1/BRCA2 mutation, where intensive surveillance and screening can be performed by yearly mastography and MRI at age 30 years [98, 101].

## Moderate-penetrant genes

### ATM

The ataxia telangiectasia (ATM) gene (on positions 22 and 23 of chromosome 11) [102] plays a key functional role in cells’ response to DNA damage. It encodes a PI3 K-related protein kinase that is involved in activating cellular responses to DNA double-strand breaks via phosphorylation of key factors in the DNA damage-response pathway [103].

Individuals harboring one copy of ATM associated with gene deletion in each cell are at a high risk of BC [7, 104]; subsequently, cells with missed one copy of this gene are capable of providing half the normal amount of ATM protein, that lead to prevention of proper repair of DNA damage, resulting in occurrence of mutations in other genes [7].

Germline mutation in the ATM gene has been estimated to attribute to a prevalence of around 0.5% [104, 105]. It was estimated that heterozygous carriers of ATM mutations have a twofold higher breast cancer risk. An increasing body of evidence indicated that this risk can be elevated in women under the age of 50 years [69, 106]. Women harboring the ATM c.7271 T as a pathogenic mutation may confer higher BC risks [107], although it can be debatable because of limited evidence [108, 109]. There is increasing evidence of radiosensitivity in ATM mutation carriers regarding mice models and in vitro experiments. ATM mutations are likely linked to an increased level of sensitivity to platinum-based antineoplastic drugs [110, 111]. In addition, inhibition of DDR kinases, such as ATM and ATR, has been revealed to increase the response to ionizing radiation in some kinds of cancer including ovarian and cervical carcinoma in vitro, while was not capable of increasing the response to platinum drugs [112]. Furthermore, BRCA1 is markedly involved in homologous recombination in primary mouse somatic cells, but not the ATM [113]. It is not worthy that further clarification will require systematic and in-depth understanding of platinum sensitivity and ATM-aberrant cancers.

Contradictory results have been reported regarding radiotherapy in carriers of a pathogenic ATM variant where emerging data has indicated increased toxicity, and other evidence indicates clinical benefits [114]. However, high risk has been reported for contralateral breast cancer in subjects with ATM missense mutations who underwent radiotherapy, indicating a lack of proper treatment with radiotherapy [115]. Targeting ATR can be taken in to consideration, where single-agent ATR inhibitors revealed to be potential therapeutic agent for mantle cell lymphoma with ATM-deficiency [116]. Accumulating evidence demonstrates that ATR-checkpoint kinase 1 (Chk1) pathway can be considered as potential therapeutic strategies for ATM-deficient cancers. VE821, VE822, and AZD6738 are ATR inhibitors which are used in preclinical studies [117, 118]. AZD6738 is an analogue of AZ20 that exhibited single-agent anti-tumor activity across cancer cell lines in ATM-deficient [119, 120]. AZD6738 is currently applied in subject suffering from solid tumors as a single-agent and/or in combination with palliative radiotherapy (NCT02223923). Single agent activity of PARP inhibitors has been demonstrated in ATM-deficient tumor cells [121, 122]. Based on available data, the loss of both ATM and p53 function resulted in an increased level of PARP cytotoxicity [123]. However, to the best of our knowledge, PARP inhibitors have not been clinically investigated in ATM-deficient tumors. Inhibitors of other potential DDR targets including PARP, CHK1/2, WEE1, and DNA-PKcs are considered as novel therapeutic approach for development of ATM and ATR inhibitors with the potential to improve cancer outcome [124].

Chk1 inhibitors are currently being evaluated in clinical studies, which help to fulfill the potential of the anti-metabolite therapies and cytotoxic chemotherapy and anti-metabolite therapies [125]. In addition, Chk1 inhibitors are in clinical development, for which chemosensitizing activity and monotherapy have been indicated in ATM [110]. V158411 single agent or in combination with cytotoxic chemotherapy are considered as clinical therapeutic strategy for triple-negative breast cancer [125]. MK-8776 (SCH-900776) as Chk1 inhibitor has been suggested to contribute to an increased level of cell death induced by chemotherapy [110, 126]. Furthermore, antileukemia activity of SCH900776 has been revealed in a phase I trial. In preclinical studies, PF-00477736 and AZD7762 have been proposed as chemotherapy sensitizers in cancer cells or xenografts [127]. Some clinical trials for ATM-deficient breast cancer is documented (ClinicalTrials.gov Identifier: NCT02264678; NCT03565991).

### CHEK2

CHEK2 gene at position 12.1(22q12.1) [128] provides instructions for making checkpoint kinase 2 proteins as a tumor suppressor. This protein is implicated in the DNA damage signaling pathway [129].

CHEK2 plays a crucial role in regulation of p53 function and BRCA1, and it stops the cell from dividing and a single DNA building block at nucleotide sequence 1100delC can led to abnormality.

A particular germline mutation, CHEK2c.1100delC, has been found to be not only linked to enhanced risks of BC [130] and male breast cancer [131], but have also been related to response to therapy [132]. While it seems to occur with a frequency of 0.5–2% in European individuals [133]. A previous study indicated that women with homozygous CHEK2c.1100delC mutations are at risk breast cancer, sixfold higher than heterozygotes [134]. CHEK2p.I157T variant is linked to lower breast cancer risk (∼ 1.5) [135]. Moreover, mutations including S428F and CHEK2del5567 were identified in other populations [136]. It has been reported that CHEK2.S428F increase risks of female BC by about twofold [130]. In primary breast cancer, CHEK2 or TP53 mutations have been revealed to be involved in a functional pathway linked to resistance to epirubicin and anthracycline-based chemotherapy [132, 137]. Furthermore, it has been suggested that CHEK2 H371Y mutation carriers may represent favorable response to neoadjuvant chemotherapy [138]. However, contradictory findings have been achieved in response to endocrine therapy and/or adjuvant chemotherapy [139–141]. To the best of our knowledge, information on the effectiveness of systemic therapy for breast cancer patients with CHEK2 mutations are limited [141]. Further development will require comprehensive and in-depth understanding of the CHEK2 1100delC-associated breast cancer to provide further personalized therapy.

### PALB2

PALB2 was known as a BRCA2-interacting protein that localizes BRCA2 during homologous recombination and double-strand break repair, which is located at chromosome 16p12.2 [142]. The PALB2 and BRCA2 proteins are capable of helping the regulation of the cell growth, and division of cell, as well as are as tumor suppressors.

Homozygous mutations in PALB2 are linked to Fanconi’s anemia, while heterozygous mutations in PALB2 are related to developing of breast cancer risk in women with a clear family history of breast cancer [70, 143].

PALB2 loss-of-function mutations have been detected to attribute to a risk of 0.6 to 3.9% in families with a history of BC among people from many countries. Despite PALB2 variants are rarely identified in 1–4% of BRCA negative families, but increasing evidence suggests its increased risk for breast cancer as compared to BRCA2 mutations [3, 144]. However, PALB2 has been recently emerged to be a moderate-penetrant gene for breast cancer susceptibility.

Gene mutation in PALB2 increases breast cancer risk about twofold [145, 146]. PALB2 mutant genes were also found in European families with a history of breast cancer [146].

Antoniou et al. (2014) investigated PALB2 mutations by BRCAx BC study. They indicated that heterozygous PALB2 mutations carriers by age of 70 are developing BC as high as 35%, where age- and family history-dependent BC risk has been generally revealed in the population [147]. The higher rate of PALB2 mutations indicates the high risk of breast cancer in the carriers. Therefore, PALB2 mutations can be genetic markers for the clinical diagnosis and prognosis of BC because of their higher rates.

The ample evidence suggests that PALB2 is involved in DNA damage repair via the FA/HR pathway, where it plays a role as a switch panel for triggering the repair of DSB via HR [148–150]. It has been indicated that regulation of PALB2 phosphorylation, ubiquitylation, and homodimerization are involved in mediating this control [150–152]. Inhibiting these signalizations can be considered as an approach for sensitizing cells to DNA damaging agents, which provides an opportunity to develop a putative target, PALB2, for accessing cancer treatment [150].

It is worth noting that determination of the full spectrum of PALB2 mutations is required in familial breast cancer, where larger case-control studies can reveal the implication of PALB2 mutation. Further development will need in-depth understanding of the functional properties of the variant spectrum for determining the pattern of PALB2 and defective DNA repair in genesis and cancer treatment [153].

ATMs and CHEK2 are in dire need of prospective information to provide strong evidence for developing risk management and therapeutic strategies.

PALB2 mutations can lead to disease progression, while its modulation is potentially related to progression of breast cancer, showing its role in preventing tumorigenesis [150]. A growing body of evidence suggests that PALB2 functions as a tumor suppressor, which is being explored as targets for development of therapies strategies against cancer. The “synthetic lethality” offer a promising opportunity to develop novel therapy in terms of personalized medicine in cancer, such as PARP inhibitors [154]; nonetheless, PALB2 advantages for development of a specific therapeutic strategy such as PARP inhibitors is still not clear [148, 150, 155].

### BRCA1-interacting protein 1 (BRIP1; FANCJ/BACH1)

BRIP1 has been defined to interact with the BRCA1, which can be colocalized with BRCA1 at sites of DNA damage, and helps in repairing damaged DNA [156]. BRIP1 gene is located at 17q22.2 near the BRCA1 locus [157]. BRIP1 interactions related to BRCA1 function suggested that BRIP1 have attracted the attention as a candidate tumor suppressor gene.

The frequent breast carcinomas (wild-type) of BRCA genes were documented but exhibit allelic losses at the 17q21-q22 region which indicates that this chromosomal region may be as a breast cancer predisposing gene. On the other hand, BRIP1 has been described to be biallelically inactivated in patients with Fanconi anemia (FA). Recently, biallelic defects in PALB2 have been revealed to be involved in FA subtype N [143, 158].

Mutation in one copy of the gene disturbs function of BRIP1 protein [159], which loss of function or missing of this marker can lead to lack of interaction with the BRCA1 protein, leading to failing the repair of the damaged DNA [160].

Previous researches reported that the inherited mutation in the BRIP1 gene is associated with the increased risk of breast cancer [161, 162]. In addition, in a study by Seal et al. (2006), truncating mutations were identified in breast cancer families, although there are reports of higher risks in some families [163]. BRIP1 germline mutations are also associated with an increased risk of ovarian cancer [164]. However, the effect of germline BRIP1 mutations in the risk of breast cancer is still controversial [165].

Studies suggest that the germline BRIP1 mutations can be considered as a moderate risk for ovarian cancer [166–168], and prophylactic surgery is taken into consideration for patients carrying BRIP1 mutation [165, 169]. While there is some evidence that truncating variants in BRIP1, especially p.Arg798Ter, are not substantially attributed to the development of breast cancer risk [166]. There is no consensus on the role of truncating variants in BRIP1 in developing breast cancer, where no large systematic studies have been performed for providing clear evidence. It is noteworthy that large systematic studies would be needed to estimate the reliable risk association with genetic variants.

### PTEN

Phosphatase and tensin homolog protein (PTEN) is a lipid phosphatase that is encoded by the PTEN gene. This gene was identified as a tumor suppressor with such functions, including regulation of cell cycle, apoptosis, and metastasis [170]. Alterations in the cell cycle regulation and a defective apoptotic response occurs in cell lines with mutations on PTEN.

Mutation or inactivation of PTEN gene had occurred up to 30% of breast cancers that can led to hyper-activation of the PI3K/Akt signaling pathway. Germline mutations in PTEN are the cause of Cowden’s disease and Bannayan–Zonana syndrome, which is described as a high risk of developing breast cancer. Affected individuals are at risk up to 50% for breast cancer [134] and also 20–50% of affected females [171].

It has been defined that cytoplasmic PTEN is capable of playing a primary role in regulating PI3K/AKT pathway as a negative regulator, while nuclear PTEN depicts tumor-suppressive function in a phosphatase-independent manner (e.g., apoptosis, cell cycle arrest, DNA repair, and regulation of chromosomal stability [172–174].

PTEN plays central role in promoting tumorigenesis, where loss of PTEN has been indicated to contribute to the resistance to cancer therapy. Preclinical findings indicated that AKT/mTOR pathway may be a good target for preventing growth of PTEN-deficient tumors. As a matter of fact, agents targeting PI3K or downstream may be a valuable source of therapeutic targets for preventing PTEN-deficient cancers, while agents acting at the level of signaling nodes upstream of PI3K Cannot be effective [174].

Knockdown of PTEN has been found to engage ErbB3 receptor tyrosine kinase activity and insulin-like growth factor-1 receptor signaling by their activation and also lead to increased level of PI3K and AKT activities, resistance to the anti-estrogens tamoxifen and fulvestrant, and cell proliferation, as well as hormone-independent growth [174, 175].

A progressing body of evidence proposes that PTEN deficiency can be liked to a decrease in sensitivity of RTK inhibitors, while more clarification is required for confirming this claim. As previously indicated, PTEN loss has been found to be related to unfavorable response in trastuzumab-treated breast cancer patients [176, 177].

Contradictory findings have been reported for PTEN, where its loss was found to be linked to better response in lapatinib-treated breast cancer patients, followed by trastuzumab [177]. On the other hand, in a phase III trial (NCCTG N9831, adjuvant trastuzumab was found to be beneficial for subjects with HER2-positive BC carrying PTEN mutation [178].

Much of the research into breast cancers can be focused on functions or mechanisms of trastuzumab anti-tumor action in patients with PTEN deficiency, where PTEN status has been indicated to be related to response of trastuzumab, an anti-HER2 monoclonal antibody, for developing therapeutic strategies against breast cancer.

In this regard, it has been revealed that pertuzumab binding to the extracellular domain of HER2 can be capable of blocking ligand-induced HER2/HER3 dimerization [179, 180] and can result in induction of antibody-dependent cellular cytotoxicity (ADCC) [181].

Many researches have focused on effect of PI3K/AKT/mTOR pathway inhibitors in patients suffering from advanced HER2+ breast cancer. BEZ235 as PI3K/mTOR inhibitor in combination with trastuzumab revealed to be capable of exhibiting a favorable safety profile in patients with HER2-positive metastatic breast cancer and PI3K pathway alterations. PTEN and PIK3CA status was not found to be linked to responses of PI3K/mTOR inhibitor BEZ235 and Buparlisib (PI3K inhibitor) in individuals with HER2+ advanced BC with metastasis [182, 183].

Epidermal growth-factor receptor (EGFR) antibody and EGFR inhibitors (e.g., cetuximab or panitumumab) have been used for developing a promising strategy for the colorectal cancer therapy in EGFR-expressing tumors (i.e., NCT01283334, NCT00522665, NCT01719380, NCT01252628, NCT01256385) [184–187].

P110β catalytic isoform of PI3K (class IA) has been demonstrated to be involved in driving phosphatidylinositol signaling in PTEN-deficient cancer cells, where further development will need clinical investigations and in-depth understanding of the molecular mechanisms to develop therapeutic option such as p110β-specific inhibitors (NCT01458067). TORC1 inhibition has been shown to be favorable efficacy for PTEN hamartoma tumor syndrome [188].

Further development will need in-depth understanding of the PTEN deficiency beyond the PI3K pathway, and genome instability as well as the effect of PTEN deficiency in modulating drug sensitivity and resistance. Table 3 summarized clinical trial cancers as new therapeutic approaches for patients with PTEN deficiency.

**Table 3:** Clinical trials cancers for treatment of patients with PTEN-deficient. Tables and data are adopted from clinicaltrials.gov. (https://clinicaltrials.gov/)

Status of trial	Conditions	Interventions of clinical trial	Study type	Study completion time
Recruiting	• PTEN mutation and PTEN hamartoma Tumor Syndrome (PHTS)	• Drug: RAD001 and placebo	Interventional, phases 1 and 2	2019
Recruiting	• Advanced solid tumors with PTEN or PIK3CB mutations • Anatomic stage III BC AJCC v8 and anatomic stage IIIA BC AJCC	• Drug: Docetaxel, PI3Kbeta and AZD8186 inhibitor • Biomarker evaluation	Interventional, phase 1	2021
Completed	• Cowden’s disease • PHTS	• Fludeoxyglucose F 18 as radiotherapy • Drug: Sirolimus	Interventional, phase 2	2012
Recruiting	• Cancer and solid tumors with AKT1, 2, 3 genetic changes, activating PI3K mutations, PTEN mutations	• Drug: ARQ 751	Interventional, phase 1	2019
Active, not recruiting	• Advanced cancers with PI3KCA mutation, Positive/PTEN under-expression	• Drug: Pazopanib and Everolimus	Interventional, phase 1	–
Not yet recruiting	• Metastatic BC with AR+ and PTEN+	• Drug: Alpelisib (BYL719), and Enzalutamide (androgen receptor inhibitor)	Interventional, phase 1	2021
Completed	• Evaluation of tumor suppressor gene PTEN endometrial neoplasms	• Behavioral: questionnaire • DNA testing/tumor-genetic markers	Observational study	2011
Terminated	• c-MET inhibitor; PI3K inhibitor, PTEN mutations, homozygous del. PTEN neg.	• Drug: INC280 and Buparlisib	Interventional, phases 1 and 2	23, 2016
Recruiting	• Metastatic melanoma and PTEN loss, malignancy of skin • Metastatic melanoma	• Drug: GSK2636771 and Pembrolizumab	Interventional, phases 1 and 2	2021
Recruiting	• Carcinoma, squamous cell of head and neck with PI3KCA mutation and/or PTEN under-expression	• Drug: Copanlisib and Cetuximab	Interventional, phases 1 and 2	2020
Completed	• Advanced solid tumors with PTEN deficiency	• Drug: GSK2636771	Interventional, phase 2	2016
Recruiting	• Advanced cancers with somatic mutation in BRCA1/2 • Mutations/or deletions in PTEN /or PTEN under-expression	• Drug: Talazoparib Tosylate	Interventional, phase 2	2020
Recruiting	• BC with PIK3CA/AKT1/PTEN-alteration • TNBC	• Drug: Ipatasertib, paclitaxel and placebo	Interventional, phases 1 and 2	2021

### MRE11-RAD50-NBS1 (MRN)

The highly conserved MRE11–RAD50–NBS1 (MRN) is known as a complex that plays an important role in DNA double-strand break regeneration (DSBs) via homologous recombination and on-homologous end-joining (NHEJ) pathways as well as in telomere maintenance, DNA replication, and cell cycle checkpoints [189]. Dimers of the proteins encoded by MRE11A, RAD50, and NBN are implicated in forming this complex [190–195].

Deregulations of DNA damage signaling, checkpoints, and repair pathways has been revealed to be linked to cancer progression and genotoxic therapies response such as ionizing radiation and diverse chemotherapeutics.

Based on the data presented in the literature, the ATM/MRN-coordinated DNA damage response network can be usually activated in early stages of different kinds of cancer, which there has been increasing evidence that suggests the DNA breakage and oncogene-induced replication stress are partial. Therefore, DNA damage response network has been considered to be potentially a barrier against cancer [195].

Individuals harboring biallelic mutant MRN complex gens (only one gene) are sensitive to ionizing radiation deficit in DNA DSB repair, and genome instability [191]. Germline homozygous mutations of individuals in NBS1 have been defined as Nijmegen breakage syndrome (NBS), for which an increased risk of cancer can be regarded. Among three genes in the MRN complex, the inherited NBN gene change has the strongest evidence to act as an intermediate-risk breast cancer gene. It has been indicated that heterozygous germline mutations of MRN complex can be potentially capable of developing different types of cancers such as breast and ovarian cancers such as BC and ovarian cancer (OC) [192, 193, 196], indicating that carrier of a deleterious mutation in NBN may be capable of increasing the risk about two- to threefold [192]; however, its role is controversial. Moreover, an increasing body of evidence indicated tow mutations in MRE11A gene can lead to under-expression of all MRN proteins in non-BRCA1/2 BC families [197]. MRN defects have been described to be associated with chromosome instability. On the other hand, an increasing body of evidence supports that MRN are under evolutionary pressure to coordinate cellular network responses to DNA damage associated with mutation, cancer progression, and apoptosis [198]. There is a hypothesis that RAD50 can act as intermediate-risk BC and/or pancreatic cancer gene, but has no strong evidence in supporting this hypothesis [193–195].

Downregulation of the MRN and its function defect has been found to be capable of resulting in DNA damage, higher propensity for cellular destabilization, and cell transformation in tumor-development. However, controversial data are presented in the literature using different cell lineages, where a complex association has been found between expression level of MRN and carcinogenesis. MRN overexpression and its components have been suggested to be linked to worse outcome and chemoradiation resistance through its vital role in DNA DSB repair while others studies have published contradictory findings [199–203]. MRN expression has been suggested to be a key influential factor in cancer cells response to chemotherapy, radiation therapy, and the level of apoptosis [199]. Recently, homologous recombination repair of double-strand DNA breaks has attracted researcher focus as a target to develop an appropriate therapeutic strategy, e.g., PARP inhibitors for BRCA1/2-deficient cancers [204, 205].

A progressing body of evidence reveals that MRN complex mutation-related HR deficiency may be capable of sensitizing cancer cells to PARP inhibitors therapy, and might thus be applicable for providing a predictive biomarker of PARP inhibitor-based therapy [206–208]. Nonetheless, the important role of the MRN complex and its components has not been fully understood in clinical trial to determine molecular mechanisms that underlie regulation of the MRN and its influence in cancer progression, prognosis, and chemosensitivity.

### RAD51

RAD51 and the family of RAD51-related genes are implicated in encoding proteins that play their role in interaction with a set of proteins such as BRCA1, BRCA2, and PALB2 and p53 for repairing the damaged DNA in cellular damage-sensing and cell cycle checkpoint pathways [209, 210]. RAD51 recruitment to break sites and recombinational DNA repair depend on the RAD51 paralogs [211]. Mutations in RAD51 can led to loss of RAD51 focus formation in response to DNA damage [212]. RAD51C is considered as an important part of the DNA double-strand repair; its biallelic mutations were observed in ~ 1.3% of BRCA1/2-negative BC and/or OC families [213].

There are many studies showing the presence of mutant RAD51C mutations in breast cancer and/or ovarian cancer families, indicating RAD51C as a cancer-predisposing gene [214, 215]. It is also linked to both Fanconi anemia-like disorders. Moreover, it is worth noting that the other RAD51 paralogs, such as RAD51D and RAD51L1, may be related to breast and/or ovarian cancer risk [216], although it needs to be proven by clinical significance of these findings that is unknown.

It has been identified that 1.3% of BC and OC families had RAD 51C mutations, where emphasized on a rare mutations of BC cases in families [216]. Additionally, Wenping et al. (2012) suggested that RAD51C mutations are rarely occurred among high-risk BC and BC/OC families [217].

RAD51 is known as a central homologous recombination (HR) implicated in HR pathway via catalyzing the strand transfer for repairing the damaged area. It is capable of forming a helical filament on single-strand overhangs that can be exonucleolitycally created on DSBs during presynaptic step [218, 219].

Dysregulation of RAD51 is capable of impairing HR and inducing aberrant genome rearrangements, genetic phenomena that can be seen in a variety of cancers [209]. However, upregulation of RAD51 has been reported in many types of tumor cells [219, 220]. It is believed that high expression level of RAD51 protein can be potentially able to develop a mechanism for compensatory alternative DNA repair, and compensating the functional BRCA1 loss involving in survival of cancer cells and tumorigenesis, e.g., metastatic progression of triple-negative breast cancer [221–223]. Targeting of RAD51 is linked to enhanced kinase signaling, indicating other mechanism linked to loss of synthetic lethality. It is noteworthy that kinase signaling in triple-negative breast cancer has been described by various rewiring phenomena as compensatory mechanisms for resistance to therapy [224–226]. Current evidence indicates that a combined targeting of RAD51 p38 can be linked to inhibition of cell proliferation in vitro and in vivo. Furthermore, depletion of RAD51 has been found to be linked to enhanced ERK1/2 and p38 signaling. Targeting of RAD51, PARPi, and p38 may provide a therapeutic approach for TNBC, without a synthetic lethal resistance, through inducing pressure on the pathways of DNA repair [227].

The role RAD51 in breast carcinogenesis has been suggested by previous studies. Some investigations indicated concomitant under-expression of BRCA1 and upregulation of RAD51 in sporadic invasive ductal breast cancer. Contradictory findings reported under-expression of both BRCA1 and RAD51 in BC cell lines and BC cells [209, 220, 228].

Based on the data presented in the literature, RAD51 has been revealed to be a key target of miR-155 both in vitro and in triple-negative BC. MiR-155 is capable of regulating DNA repair process and sensitivity to IR via repression of RAD51 in BC. As a matter of fact, miR-155 targeting of RAD51 can be mainly involved in induction of a delay in repair after IR exposure [229]. B02 has been introduced to be efficiently capable of inhibiting DNA strand exchange feature of RAD51, DSB repair, and DSB-dependent HR [230]. An increased MDA-MB-231 cell killing has been reported using B02 especially better efficacy has been achieved in combination with cisplatin. B02 was also capable of sensitizing MDA-MB-231 breast cancer cells to cisplatin in mouse xenografts. Additionally, B02 resulted in disruption of RAD51 foci formation in MDA-MB-231 cells; therefore, it has been previously concluded that B02 can be considered as an effective agent via targeting RAD51 in vivo, indicating its potential to offer a promising combination strategy for the treatment of cancer [219]. Further development would be needed to appropriately clarify the molecular mechanisms of the RAD51 and its influence in BC in order to develop new anti-cancer therapy.

### BRCA1-associated RING domain 1 (BARD1; MIM #601593)

The human BARD1 gene spread out over long arm of chromosome 2 (2q34-2q35) with an 85-kb region. The sequence and structure of BARD1 has been defined to share similarities with BRCA1, forming a functional heterodimer with BRCA1 via RING fingers of both BRCA1 and BARD1. Both BARD1 and BRCA1 proteins possess a RING finger domain at the N-terminus and two C-terminal BRCT domains; RING domains demonstrated E3 ubiquitin ligase activity, which play a vital role in targeting proteins implicated in many biological process such as cell-cycle regulation, DNA repair, and modulation of chromatin structure [231]. Ubiquitin ligases demonstrated a major role for the initiation of polyubiquitination, which are key proteins involved in degradation by the proteasome. Mutations of BRCA1 RING domain lead to breast cancer by disrupting the E3 ubiquitin ligase activity of BRCA1-BARD1 [232]. The BARD1/BRCA1 heterodimer has been found to be implicated in homologous recombination DNA repair and maintaining genomic stability, whereas defective function and/or dysregulation of either protein can be associated with genomic instability [233, 234]. Therefore, it has been revealed that BARD1 plays crucial role in maintaining genome integrity and cell viability [235].

BARD1 protein interacts with BRCA1 in DNA double-strand break repair and initiation of programed cell death [233, 236]. Based on the data presented in the literatures, occurrence of missense mutations in the BRCA1 RING-finger domain are capable of preventing the formation of heterodimers, demonstrating very slightly deleterious mutations [233, 235, 237].

It has been shown that missense mutations in the BRCA1 RING-finger domain are capable of preventing the formation of heterodimers, demonstrating very slightly deleterious mutations [238]. Mutant BRCA1 and BRCA2 gens are linked to emerging various hereditary BC, accounting for 1% of all BC patients with a lifetime risk of 50–85% [239, 240]. Increasing evidence suggests that BARD1 mutations is very likely to occur in patients with non-BRCA1/2 inherited breast cancer [241].

Some breast cancer-related BRCA1 missense mutations disturb the function of the BRCA1/BARD1 complex. In a previous study, missense BARD1 variant, Cys557Ser (rs28997576), has been revealed to be highly upregulated in breast cancer families [242, 243]. Missense mutations in BRCA1 (e.g., p.Cys61Gly) has been shown to be capable of distracting the BARD1/BRCA1 interaction, thus leading to breast cancer [244].

Previous studies indicated that mutations in the RING finger domain of BRCA1 are capable of making disturbance to BRCA1/BARD1 interaction in BC [245] and the presence of BARD1 missense mutations in subjects suffering from BC revealed that BARD1 gene tendency to participate in BRCA1 mediated tumor suppression [246].

Another study has reported a protein truncating mutation (p.Glu652fs) in BARD1 gene among family with great risk and also a homozygously deleted BARD1gene in a BC family [247, 248]. Previous studies identified BARD1 mutations in high-risk families [249].

Based on the data presented by large whole exome sequencing, BARD1 have merged among the genes with somatic alteration at low level [250].

BRCA1 has a tumor suppressor role, which is likely to be under control via the BARD1-BRCA1 heterodimer [231], and ubiquitination-dependent pathways are involved in BRCA1 degradation [251]. As a matter of fact, BRCA1 protein plays its functional role via ubiquitin ligase activity [12], thus is capable of affecting DNA repair, and cell control mechanisms, as well as mitosis, via targeting proteins for degradation [232, 252, 253].

A set of data demonstrated that somatic mutations and/or predisposition gene silencing variants can be associated with dysregulation of full-length BARD1 (FL BARD1), which can be taken into attention as an early hit of BARD1 tumor suppressor function. Furthermore, cancer-related BARD1 isoforms have been shown to be capable of antagonizing the functions of FL BARD1 tumor suppressor, resulting in genetic instability, DNA repair-deficiency, and loss of cell cycle regulation and increased cell proliferation [233].

Cancer-related isoforms of BARD1, except for FLBARD1, can result in proliferation arrest in vitro [232, 254, 255]. Current evidence suggests that protein interaction, pro-proliferative pattern, and localization of BARD1 isoforms are found to be highly different as compared to FL BARD1, indicating that isoforms obtained tumor-promoting functions, while losing tumor suppressor functions of FL BARD1 [232, 254]. Furthermore, FL BARD1 has been shown to play a functional role in turnover of the mitotic aurora kinases. Aurora kinases have been described to be upregulated in various cancers leading to genetic instability, thus its expression level can potentially be considered as biomarkers for predicting response to aurora inhibitors [235, 256, 257].

BRCA1 mutations have been reported to be associated with resistance to both PARPi and platinum as therapeutic approach. BARD1β has been introduced to evaluate the suitability of colon cancers for homologous recombination targeting using PARPi [233, 258–260]. Further investigations are necessary to explore the role BARD1 function in cancer in the future as well as to determine the efficacy of PARPi in BARD1 mutated gene with its defective function. PARPi is explained to be involved in inducing multiple double-strand breaks therefore it can be highly linked to repairmen of cell death, when double-strand breaks in identified BRCAs (1 and 1) mutations are repaired at low level in cancer [235, 261].

### EGFR, ERBB2, and activating ERBB2 (HER2) mutations

There are four members including EGFR, ERBB2 (HER2), ERBB3, and ERBB4 in the family of epidermal growth-factor receptors (EGFRs). These receptors mediate normal cell proliferation and cell survival via two major signaling pathways: Ras-Raf-MAPK and PI3K/Akt/mTOR. Considering breast cancer, high levels of the EGFR and c-erbB2 correlate with poor prognosis because of a high incidence of metastases and intrinsic resistance [262, 263].

The EGFR is robustly expressed in TNBC, and indicated to be strongly related to poor prognosis. Moreover, overexpression of the EGFR has been found in 20–25% of BCs and was correlated with poor prognosis and worst survival rates of BC subjects. It is worth noting that the expression of transforming growth factor, and TGF-α, and another EGF family member, named amphiregulin, is the most commonly increased in BCs [264]. High copies of ErbB2 led to overexpression of the ErbB2 receptor protein. As result, high amount of ErbB2 receptor protein induces continuous signals for rapid growth of cells and cell division which result in developing cancerous tumors. The non-receptor tyrosine kinase Src, PI3K, and MAPK pathways mediates ErbB2 increased proliferation, survival, motility, and invasion. ErbB2 plays an important role in human malignancies [265]. The erbB2 expression was detected to be increased in 20–30% of human BCs ErbB2 (HER2) positive BC strongly related to poor prognosis that result in emerging metastases nature of cancer and intrinsic resistance to endocrine and routine chemotherapy [266, 267].

The HER pathway has been defined to be related to the pathogenesis of breast cancer, which are particularly overexpressed in subjects with HER2 mutations [268]. It has been previously estimated that wild-type HER2 overexpression is linked to 20–30% of breast cancers; however, another study indicated that HER2 mutation is contributed to 6% prevalence for breast cancer subjects [269].

Many investigations have further described the occurrence of HER2 mutations in many kinds of solid tumors (e.g., breast, non-small cell lung, colorectal, and bladder cancers) by development of the Cancer Genome Atlas projects [270–272], where their highly recurrent nature and their presence in the kinase domain and/or extracellular domain mutations at residues 309–310 have been revealed [269, 273, 274]. In addition, it has been previously demonstrated how both oncogenic transformation of breast epithelial cells and tumor growth enhancement can be developed by HER2 mutations [269, 273], thus leading to tumorigenesis. A host of approaches are being evaluated HER2 mutation positive cases, where resistant to the reversible HER2 inhibitor lapatinib has been revealed; on the other hand, neratinib was reported to be beneficial for treating the HER2 mutation positive subjects [269, 275–278]. A study demonstrated that seven HER2 mutations activated the protein including V842I, R896C, G309A, D769H, D769Y, V777 L, and P780ins, leading to providing benefit from existing HER2-targeted drugs (neratinib) for breast cancer patients with refractory HER2 positive [269].

Neratinib and/or neratinib-based combinations have been considered for providing therapeutic strategies in terms of HER2-mutated BC. As indicated in Table 4, different clinical phases of neratinib monotherapy and neratinib-based combination have been developed for treating the HER2 mutation positive patients by neratinib (HKI-272) for which there is an increasing evidence of encouraging therapeutic efficacy.

**Table 4:** Clinical trials for treating the HER2 mutation positive patients by irreversible pan-ErbB tyrosine kinase inhibitor (*Neratinib*; *HKI*-*272*). Tables and data are adopted from clinicaltrials.gov. (https://clinicaltrials.gov/)

Status of trail	Condition	Interventions of clinical trial	Phase	Completion time
Completed	• BC • Advanced solid tumors	• Drug: Neratinib and vinorelbine	Phases 1 and 2	2018
Completed	• Breast neoplasms	• Drug: Neratinib	Phase 2	2018
Completed	• Advanced BC	• Drug: HKI-272 and trastuzumab	Phases 1 and 2	2018
Completed	• Advanced BC • Advanced solid tumors, breast neoplasms	• Drug: HKI-272 and paclitaxel	Phases 1 and 2	2018
Active, not recruiting	• BC	• Drug: Neratinib and placebo	Phase 3	2020
Completed	• BC	• Drug: Neratinib and capecitabine	Phases 1 and 2	–
Recruiting	• BC	• Drug: HKI-272, Capecitabine, Ado Trastuzumab Emtansine	Phase 2	2023
Completed	• Breast neoplasms	• Drug: Neratinib	Phase 1	2007
Completed	• BC	• Drug: Neratinib, trastuzumab and paclitaxel	Phase 2	2018
Completed	• Advanced BC • BC	• Drug: Neratinib, lapatinib and capecitabine	Phase 2	2018
Completed	• BC	• Drug: Temsirolimus and neratinib	Phases 1 and 2	2016

### DIRAS3/ARHI (DIRAS family, GTP-binding RAS-like 3)

The DIRAS3 gene is defined as a member of Ras genes. It has been mapped to the 1p31*.*3 [276]. ARHI is capable of encoding a small GTP-binding protein of Ras family that plays its role as a tumor suppressor gene in OV and BCs [276, 279]. Two copy of DIRAS3 genes are inherited from parents, and its expression from the paternal allele has been described in all normal cells, namely genomic imprinting. Based on the current evidence, ARHI re-expression in breast and ovarian cancer cells has been defined to be capable of activating JNK pathway, leading to apoptosis and inhibition of tumor progression and metastasis [280–282]. Ample evidence supports that deregulation of DIRAS3 function can be caused by a “single hit” over period of carcinogenesis [276]. This increases the susceptibility for developing of BC. To the best of our knowledge, mutations of ARHI have not been revealed for the coding and promoter areas [283]. DIRAS3 protein as an imprinted tumor suppressor shows normal expression level in breast epithelial cells, whereas is under-expressed in more than 70% of BCs. Its downregulation is associated with developing of tumor in BC [279].

STAT3 play a key role in regulating the methylation status of many kinds of tumor suppressor genes, leading to their downregulation such as ARHI. It has been indicated that ARHI is capable of blocking nuclear transport of STAT3 via interacting with STAT3 [280, 284]. On the other hand, it has been revealed that ARHI is capable of competing Ran proteins, and is involved in forming a complex with importinβand, thus leading to prevention of the nuclear transport of STAT3. A progressing body of evidence reveals that downregulation of ARHI plays a key role in regulating of machinery for nuclear transport of STAT3, leading to upregulation of oncogenes in tumor. In this regard, further in-depth understanding of interacting mechanism of ARHI with STAT3 and importinβcan are needed to provide new molecules that can be capable of mimicking the interactions of ARHI protein with these proteins [280].

Previously, ARHI re-expression has been reported to be implicated in inducing autophagic cell death in breast cancer cells, and is able to increase the growth inhibitory effect of paclitaxel through promotion via some biological process, e.g., apoptosis, autophagy, and G2/M cell cycle arrest [283].

### Androgen receptor

The human androgen receptor (AR) is a member of the nuclear receptor superfamily, which is involved in hormone action. The AR gene on chromosome Xq11-12 is responsible for encoding a 110 kDa cytoplasmic protein. The AR gene is located on chromosome Xq11-12 and encodes a 110 kDa cytoplasmic polypeptide. AR can also be activated through a non-transcriptional/non-genomic mechanism that does not need. AR receptor is widely expressed in 70–90% of all breast cancers and has been suggested to be effective as therapeutic target in ER-negative breast cancers, because it is widely expressed in the most of ER-positive breast cancers (67–88% of cases) with apocrine differentiation, which is related to a reduction in mortality [285–288].

Concerning the matters about ER-negative metastatic breast cancer, AR expression was identified in 12–50% of cases [289, 290], which is linked to lower rate of survival [291].

There is an apolymorphic CAG trinucleotide repeat in the AR gene. Based on the evidence, the CAG repeats are usually occurred about 8 to 35 times in normal individuals [292]. It has been reported that breast cancer risk may be influenced by indirect effect of androgens via their conversion to estradiol, or by binding with AR to promote or by stopping breast cell growth, or even by binding to ESR1 and inducing proliferation in breast cells [293]. Several specific mutant ARs have been detected among BC patients [294, 295] in female breast cancers. Several studies have revealed the CAG polymorphism, with CAG repeats in the gene, which increased risk of developing BC in female gender. However, the correlation between this polymorphism and BC has yielded conflicting results [296]. Some studies suggest relationship of short or long CAG repeats with BC [297–300]. On the other hand, discrepant results have also reported no association [301, 302]. Nevertheless, the involvement of endogenous androgens in developing BC is unclear.

There is not a clear association between AR signaling and breast cancer risk. It has been emerged that AR signaling is involved in breast cancer, but dichotomous association of AR with ER status and molecular subtype. Conflicting results are available in the literature, when current evidence suggests different prognostic role of AR among molecular subtypes, e.g., ER-negative BC and ER-positive breast cancer [303, 304]. For instance, AR has been recognized that AR in ER-negative breast cancer is able to bind a nucleus ligand, inducing cell proliferation while, however, its activity remains equivocal in ER-negative breast cancer. A phase II trial indicated a crosstalk between AR and ERBB2 signaling for elevating cell proliferation in TNBC [305]. AR has been controversially suggested to be capable of showing anti-proliferative activity in ER-positive tumors, and its status was found to be inked to favorable outcome in ER-positive breast cancer [306–308]. Overall, AR has been defined to be linked to a transcription factor that is capable of regulating genes implicated in opposite, cellular processes; it was found to be able to induce or inhibit cell proliferation and apoptosis, based upon the concurrent signaling pathways activated [309–312]. AR-blockade in AR-positive TNBC has been revealed as previously reported by phase II clinical trials, e.g., clinical trial investigating abiraterone acetate plus prednisone (UCBG 12-1), [305], trial evaluating enzalutamide (MDV30100-11) (Traina et al. 2018), and trial of bicalutamide [313].

The anti-androgen agents (e.g., bicalutamide, enzalutamide) provided novel approach for development of therapeutic strategies in advanced breast cancer expressing AR. However, further development will have needed in-depth understanding of mechanisms underlying AR effect in the pathogenesis of BC. Increasing body of evidence indicates that AR is able to interact with a set of signaling pathways including MAPK pathways, cell cycle and PI3K/AKT/mTOR, or Ras-dependent pathways and many crucial molecules, such as pioneering factors (FOXA1), PTEN, BRCA1, and BRCA2 are implicated in carcinogens and progression of TNBC via interaction with AR [314]. Targetable agates such as Src, PI3-K, and/or MEK inhibitors or their rational combinations with therapies based on hormone and chemotherapeutic agents are currently provided for further assessment in breast cancer [315].

### Low-penetrant breast cancer

Many low-susceptibility loci have been identified through genome association studies (GWAS). Easton et al. (2007) detected susceptibility loci in some BC (i.e., FGFR2 at 10q26, a locus on 8q, LSP1 at 11p15, TNRC9/TOX3 at 16q12, MAP3K1 at 5q11), [302]. Hunter et al. (2007) recognized correlation of fibroblast growth factor receptor 2 (FGFR2) with sporadic postmenopausal BC and also observed other susceptibility loci including TLR1/TLR6 (on 4p) and RELN (on 7q), [316]. A number of loci have been previously discovered on chromosomes via GWAS including 1p11.2 (rs11249433), 2q35 (rs13387042), 16q12 (rs3803662), 3p24 (rs4973768), 6q22.33 (rs2180341), 6q25.1 (rs2046210), 8q24 (rs13281615), and 17q23 (rs6504950), 14q24 (rs999737), as well as 5p15.33 (TERT rs2736098), etc. Increasing evidence suggests that these emerged single-nucleotide polymorphisms (SNPs) may be associated with BC susceptibility [316].

## Concluding remarks, the potential treatment model of breast cancer and current and future perspectives

GWAS and other studies revealed modifier alleles with high (e.g., BRCA1, BRCA2, TP53, PTEN, etc.) to moderate penetrance (e.g., BRIP1, CHEK2, ATM, and PALB2, etc.) among population [317–324]. These modifier alleles can be related to the malignant transformation of breast epithelial cells, where they are capable of disrupting the molecular and cellular pathways involved in regulation of many biological process [324, 325]. The current review indicated a growing body of evidence that suggests multiple genetic modifiers of the human breast cancer, of course other modifier alleles can exist. Nevertheless, the majority of these modifiers needed more clarification and in-depth understanding in terms of their mechanistic roles in biology of breast cancer.

Molecular targeted therapy and immunotherapies are attracting attention of researchers as they offer approach for providing novel targets for future therapeutic strategies.

Today, effective targeted therapies have been identified for breast cancer that is believed to effectively block various molecular pathways. Trastuzumab or Herceptin has been proposed to contribute to the favorable efficacy for neoadjuvant and adjuvant therapies in HER2-positive breast cancer [326–329].

HER2 protein targeting has been found to be the most efficient targeted therapy as its high expression is detectable in the surface of breast cancer cells, and its upregulation is linked to malignant transformation and worst overall survival of patients with breast cancer [330, 331].

Recombinant antibody of trastuzumab has been confirmed by FAD for targeting HER2-positive breast cancer, which is capable of binding juxtamembrane area of HER-2 tyrosine kinase receptor, leading to the uncoupling of the HER2/HER3 heterodimers and is consequently involved in blocking downstream signaling and cytotoxicity [331–333]. Trastuzumab-DM1 (TDM1) has been proved to be persistently capable of inhibiting trastuzumab sensitivity and resistance of HER2-positive cells lines in breast cancer [331, 334]; it has been indicated to be involved in breakdown of the blood-brain barrier. Moreover, 264RAD antibody has been provided to be used for integrin αvβ6 targeting, resulting in decreasing progression of trastuzumab resistant in HER2-positive breast cancer cells [330]. Bevacizumab or avastin has been proved to be capable of inhibiting the development of new blood vessels which are implicated in providing energy supplies such as oxygen and nutrients to cancer cells [331]. Targeted therapies for VEGF has been applied, where bevacizumab has been proved to be a therapeutic approach for metastatic breast cancer, which prolongs progression-free survival, in comparison with paclitaxel or docetaxel [335, 336].

Dual-targeted therapy using trastuzumab and pertuzumab can be another strategy in this regard.

The dual targeting of HER receptors (HER1, HER2, HER3) by applying antibody therapy may pave the way for opening a novel therapeutic approach order to overcome cetuximab resistance induced by cancer cells, therefore U3-1287 as an anti HER3 monoclonal antibody was capable of reducing proliferation via inhibiting of both MAPK and AKT pathways and diminishing signaling from three HER receptors [337].

T-DM1 plays an important role in heavily pretreated patients with HER2-positive advanced breast cancer and contributes to the breakdown of the blood-brain barrier [326].

mTOR inhibitors (e.g., everolimus, ipatasertib, and buparlisib) and CDK4/6 (e.g., palbociclib, ribociclib, and abemaciclib) have been introduced to be capable of reversing the resistance to targeted agents and endocrine agents. CDK4/6 is considered as a key role in regulating cell cycle progression and is associated with tumorigenesis in breast cancer [326].

Regarding PARP inhibitors, talazoparib is recently evaluated in phase III trial, where its binding to DNA via trapping PARP–DNA complexes has been demonstrated, indicating its potency in primary clinical practice (NCT01945775), [338, 339]. Sunitinib has been indicated to be potentially involved in suppression of angiogenesis, proliferation of tumor cells, and migration, as well as development and progression of basal like breast cancer cells [331, 340]. Everolimus, as a m-TOR inhibitor, has been proposed to inhibit cancer cells from getting energy [341]. PARP inhibitors have been also reported to attribute to a great potential therapeutic strategy for theBRCA1/2-mutated subgroup among patient suffering from TNBC [326]. Overall, novel targeted agents have been provided an opportunity for the development of targeted therapy in terms of specific molecular subtypes of breast cancer, e.g., PI3K/AKT/mTOR, PARP, CDK4/6, VEGF, multiple kinases, immune checkpoint (e.g., Keytruda [pembrolizumab], anti PD-L1 antibody atezolizumab, etc.) and HER2 inhibitors [326, 338].

However, the intra- and/or intertumor heterogeneity of the breast cancer showed its diversifying roles in the tumor initiation and progression, where it has facilitated the presence of diverse biomarkers in primary and metastatic stage of tumors as well as in single tumor, leading to difficulty in determining a favorable genetic signature for all subtypes or each subtype, particularly tumor stem cells [326]. Combination has been proposed to attribute to an increased tumor cell killing, while also rises different adverse events.

A large number of combination therapies are proposed based on the agents which are capable of targeting the PI3K/AKT/mTOR pathways, e.g., everolimus combined to HER-2 or estrogen receptor, everolimus in combination with exemestane, combination of everolimus and endocrine therapy, PI3K inhibitors combined to CDK4/6 inhibitors and endocrine therapy, etc. [243, 326, 342]

For instance, combination therapy regimens with everolimus and HER-2 or estrogen receptor inhibitors have been proposed for inhibiting mTOR pathway as future targeted therapy to reverse sensitivity of breast cancer cells to previous therapeutic approaches, thus leading to prevention of resistance mechanisms when the mTOR pathway shows hyperactivity [331, 343]. It has been demonstrated that a set of mutant genes such as EGFR, BRAF, AKT, and PI3K in EGFR and PI3K/Akt/MTOR pathways as well as pathway involving BRAF and KRAS genes are attributed to activation of downstream signaling pathways, leading to induction of high proliferation rate in cancer cells. ZSTK474 and sirolimus has been combined as inhibitor of PI3K/Akt/MTOR pathways erlotinib and gefitinib as EGFR inhibitors for favorable inhibition of PI3K and EGFR pathway on MCF10a isogenic cell linens exhibiting mutations in EGFR, PI3K, BRAF, and AKT [344].

Trastuzumab/lapatinib/hormonal therapy has been also confirmed as a combination therapeutic strategy for hormonal receptor positive and increased HER2 protein in luminal B/ HER2-enriched subtype of breast cancer [331, 345].

As mentioned, tumor cells show such as breast cancer show resistance to targeted agents in targeted therapy and though molecular alterations like mutations and epigenetic alterations in molecular pathways have been revealed, thus further development will need combination of different pathway blockades for providing favorable therapeutic outcomes [345–350].

Despite the advantages, like combination targeted therapy, there are several challenges in terms of breast cancer for providing a formula for future therapeutic strategy that be simultaneously applied against various subtypes of breast cancer in order to inhibit multiple pathways involved. Further development will require in-depth investigation of phenotypes of breast cancer subtype, different mechanisms for cell survival, and factors involved in resistance to therapy that underlie maintenance of cancer cells and its influence in each breast cancer subtype in order to provide promising and high inclusive combination therapies [331].

Researchers reported mutations in specific genes which may predispose individuals to developing certain cancers. BRCA1 and BRCA2 are among the most frequently mutated genes implicated in hereditary BC. TP53 is another cancer predisposing gene, in which significant mutation could be attributed to attribute to a TNBC. Among other high-penetrant genes, a number of rare germline mutations has been emerged that the most notable of which are PTEN (Cowden syndrome); STK11/LKB1 (Peutz-Jeghers). Cancer predisposing genes and their pathways are importance for establishment of preventative and therapeutic targets. Regarding the widespread multigene panels, whole exome sequencing is capable of providing evaluation of genetic function mutations for development of novel strategy in clinical trials. Regarding molecular findings, ample deviance indicated that a number of subgroups are sensitive to specific targeted agents (e.g., PI3K or PARP inhibitors). The mechanistic roles of majority of mutations have been poorly described in breast cancer. Further experimental studies will require in depth-understanding of molecular mechanism to empirically clarify their roles and cell type-specific functions in breast cancer biology.

Targeting the mutant proteins involved in breast cancer can be an effective therapeutic approach for developing novel drugs. Further development will need genomic profiling studies that are required to stratified tumors based on similar tumor mutational burden (TMB) or mutational signatures as compared to single driver mutation [351]**.**

This opens up opportunities for developing immunotherapy-based strategies or alternative therapies for cancers exhibiting mutations that are not actionable (i.e., RAs) or targetable. A number of studies are required to focus on addressing the unaddressed role of driver mutations in increasing genomic instability (GI) and an inverse role [351]. Further development will need in-depth understanding of the molecular mechanisms, extent and pattern, and its influence in in breast cancer at the single-cell level to provide effective therapeutic strategy.

## Abbreviations

AMPK: AMP-activated protein kinase
AR: Androgen receptor
ATM: Ataxia telangiectasia
BC: Breast cancer
CCLE: Cancer cell line encyclopedia
Chk1: Checkpoint kinase 1
EGFRs: Epidermal growth-factor receptors
FGFR2: Fibroblast growth factor receptor 2
GEMMs: Genetically engineered mouse models
GWAS: Genome-wide association studies
LOH: Loss of heterozygosity
OC: Ovarian cancer
PDX: Patient-derived xenograft
PJS: Peutz-Jeghers syndrome
PTEN: Phosphatase and tensin homolog protein
SNPs: Single-nucleotide polymorphisms
STK: Serine/threonine kinase
TNBC: Triple-negative breast cancer
WGS: Whole-genome sequencing
